# Screen-Related Parenting Practices in Mexican American Families with Toddlers: Development of Culturally- and Contextually-Relevant Scales

**DOI:** 10.3390/children12070874

**Published:** 2025-07-02

**Authors:** Darcy A. Thompson, Laura K. Kaizer, Sarah J. Schmiege, Natasha J. Cabrera, Lauren Clark, Haley Ringwood, Estefania Miramontes Valdes, Andrea Jimenez-Zambrano, Jeanne M. Tschann

**Affiliations:** 1 Department of Pediatrics, School of Medicine, University of Colorado Anschutz Medical Campus, Aurora, CO 80045, USA; andrea.jimenez-zambrano@cuanschutz.edu; 2 Adult and Child Center for Outcomes Research and Delivery Science, University of Colorado Anschutz Medical Campus, Aurora, CO 80045, USA; 3 Department of Biostatistics and Informatics, Colorado School of Public Health, University of Colorado Anschutz Medical Campus, Aurora, CO 80045, USA; laura.kaizer@cuanschutz.edu (L.K.K.);; 4 College of Education, University of Maryland, College Park, MD 20742, USA; ncabrera@umd.edu; 5 School of Nursing, University of California, Los Angeles, Los Angeles, CA 90095, USA; lclark@sonnet.ucla.edu; 6 Denver Health and Hospital Authority, Denver, CO 80204, USA; haley.ringwood@dhha.org; 7 Department of Family Medicine, School of Medicine, University of Colorado Anschutz Medical Campus, Aurora, CO 80045, USA; 8 Department of Psychiatry and Behavioral Sciences, University of California, San Francisco, San Francisco, CA 94143, USA

**Keywords:** digital media, screen device, Latino, coviewing, parental mediation, infants, toddlers, behavioral control

## Abstract

Background/Objectives: Screen-related parenting practices (e.g., restriction, coviewing) influence the way children use screen devices. Although children start using screen devices (e.g., televisions [TV], tablets) in the first few years of life, rigorously developed measures of screen-related parenting practices for parents of toddlers do not exist. The objective of this study was to develop culturally and contextually relevant survey measures of screen-related parenting practices for use in Mexican American families with toddlers. Methods: Measures were developed using an exploratory sequential mixed methods (qualitative → quantitative) approach. Mexican American mothers of toddlers (15–26 months of age) participated in semi-structured interviews. Using the interview findings, domains of parenting practices across screen device types were identified, and survey items were developed. Items were administered by phone to 384 Mexican American mothers. Analyses included evaluation of the factor structure and psychometric properties of nine domains of parenting practices and evaluations of correlations between each scale and demographic characteristics. Results: Factor analyses supported a one-factor solution for each parenting practice as follows: Restrict TV Time (8 items), Coview TV (10 items), Behavioral Regulation with TV (12 items), Restrict Mobile Device Time (8 items); Coview Mobile Devices (10 items); Behavioral Regulation with Mobile Devices (16 items), Restrict Screen Content (8 items), Allow Screen Use Around Sleep (6 items), and Allow Screen Use While Eating (6 items). The reliabilities were acceptable (Cronbach’s alphas > 0.80). Hispanic acculturation, maternal age, and child age were correlated with many of the scales of parenting practices. Conclusions: The measures developed in this study offer a way to evaluate the use and impact of screen-related parenting practices in Mexican American families with toddlers. The use of these measures will enable investigators to identify relationships among parenting practices, screen use, and child well-being, which could inform the design of early childhood interventions promoting healthy screen use in this population.

## 1. Introduction

Children start using screen devices (e.g., television [TV], smartphones) in early childhood, with the majority starting in the first few years of life [1,2,3,4,5]. This early use sets the trajectory for later use, such that those with higher duration of screen use in early childhood tend to have higher durations later in life [6,7]. Identifying factors influencing screen use in early life may therefore be critical to the promotion of healthy screen use throughout childhood.

As the main socializers of young children, parents are critical to the promotion of healthy screen use behaviors. Across all ages of children, evidence suggests that screen-related parenting practices influence the way in which children use screen devices [8,9,10,11,12,13,14,15]. Time and content restriction, coviewing/co-use, and instructive practices may be the most widely studied screen-related parenting practices [16,17]. Context-specific parenting (e.g., with sleep or eating) has received increased interest, given the relationship between screen use in these contexts with child health outcomes [18,19,20]. Finally, with the increased targeting of screen content for very young children (i.e., children less than five years old) and the ubiquitous presence of mobile devices, attention more recently has increasingly focused on parental use of screen devices to regulate child behavior in early childhood [10].

A critical limitation of the evidence on screen-related parenting practices relates to measurement. Few studies on this topic use measures that were developed using a systematic process, including evaluating the reliability or validity of the developed measures [21]. Many studies rely on one- or two-item survey questions to capture domains of parenting practices [12,22,23], limiting their ability to capture the full breadth of ways in which mothers, fathers, and caregivers are parenting regarding screen devices. Across studies that have employed rigorously developed measures of screen-related parenting practices, Valkenburg’s 15-item questionnaire for measuring TV-related parenting practices is often used [17,24,25]. Created for parents of school-age children, it measures three domains: instructive practices (discussing content together), restrictive practices (rules on content and time), and social coviewing (cowatching without active discussion). Since its publication in 1999, a handful of other measures have been developed, often building upon the Valkenburg questionnaire [26,27,28]. In a prior study, we developed scales of TV-related parenting practices for Mexican American parents of preschoolers (PPRTV) [28]. Using a mixed methods approach (qualitative -> quantitative), we adapted the Valkenburg tool to this population and age group of children. We also developed, based on interview findings, a subscale focusing on parental use of TV viewing to manage child behaviors [28]. However, as technology has progressed, there is a need to consider screen devices beyond TV, as well as the possibility that parenting practices may vary based on screen device type [24,25]. Differences in the interactive capabilities of varying devices (e.g., traditional TV has limited interactive capabilities compared to smartphones, which allow users to interact with content in numerous ways), as well as the way in which they are used, may impact parenting practices. A robust measurement of screen-related parenting practices that considers screen device type is needed to develop a deeper understanding of the ways in which parents manage screen devices for their children.

In measuring screen-related parenting practices, growing evidence also supports the need to consider child age, culture, and context [24,25,29]. Parenting practices may vary by child age, as can their impact, due to typical developmental differences across child ages [24]. In our own qualitative work, we found that parents of preschoolers reported limiting child screen use as punishment for child behavior, whereas parents of toddlers did not [9,30]. In a meta-analysis of studies on screen-related parenting, Chen et al. found that child age moderated the impact of restrictive practices on child screen time [24]. Rigorously developed measures of screen-related parenting practices specifically intended for parents of toddlers (1–2 year olds) do not currently exist. Both context and culture may also influence the practices that parents employ, as well as their impact [13,24]. For example, in the same meta-analysis, Chen et al. concluded that the impact of parental discussions with children regarding screen media content differed between Eastern and Western countries. Specifically, parental discussions with children regarding screen media content were more strongly associated with reduced screen time and reduced exposure to inappropriate content on screen devices in Eastern countries compared to Western countries [24]. Additionally, studies report varying levels of parental endorsement of specific screen-related beliefs across different countries [31,32]. Measures of screen-related parenting practices that consider child age, culture, and context are clearly needed to advance this field.

Disparities in screen use behaviors exist in Latino children and children in lower income homes compared to their non-Latino and higher income peers, underscoring the importance of focusing on child screen use in lower income Latino families [33,34]. For example, evidence suggests that Latino children spend more time each day using screen devices compared to their non-Latino white peers [33]. Recognizing the heterogeneity of the Latino population, we focused this study on Mexican American families, as 65% of Latinos in the US are of Mexican heritage [35,36]. Moreover, approximately 15% of all children in the US are of Mexican heritage [37,38].

The aim of this study was to develop measures of screen-related parenting practices for Mexican American families with toddlers. This work is part of a larger study aiming to understand social ecological contributors to early childhood screen media use in Mexican American communities. Moreover, it builds on our prior qualitative findings from interviews with Mexican American parents of toddlers and the development of a measure of TV-related parenting practices (PPRTV) for Mexican American parents of preschoolers [28,30]. This study addresses the need for age-specific and culturally- and contextually-relevant measures of screen-related parenting practices that consider screen device type. Such scales are needed to enhance our understanding of parenting and, ultimately, to inform interventions aiming to promote healthy screen use in Mexican American families with toddlers [39,40].

## 2. Materials and Methods

To support our goal of developing scales that are both culturally- and contextually-relevant to the community of Mexican American parents with toddlers, we used an exploratory sequential mixed methods approach. We started with a qualitative study, using interviews to explore parental day-to-day management of toddler screen use [30]. Findings were used to develop items reflecting screen-related parenting practices in Mexican American parents of toddlers. Items were then evaluated in the quantitative phase of this study. The Colorado Multiple Institutional Review Board approved this work, and all participants consented to participate prior to any study activities.

Item development: Items were developed through a multistep process. We conducted semi-structured interviews with Mexican American parents of toddlers, using open-ended questions and probes to understand their day-to-day management of screen use. Findings from those interviews are published elsewhere [30]. Through analysis of interview transcripts, we identified domains of screen-related parenting practices, including restriction of time and content, behavioral regulation with screens, coviewing, and two context-specific domains—screen use around bedtime and screen use while eating [30]. To develop items for each domain, we started by evaluating the relevance of items in the same parenting domains in the PPRTV [28] measure, which we previously developed for use in Mexican American parents with preschoolers. More specifically, items in the PPRTV scale, which was developed for parents of preschoolers, that reflected practices described in the interviews we conducted with mothers of toddlers were selected for use. We replaced the PPRTV wording related to TV-viewing with wording related to screens, to be more broadly defined. Then, using parental descriptions of parenting practices identified in the interview transcripts, we developed new items for each parenting domain. Finally, because interview findings suggested that parents may approach child TV use differently than mobile device use [30], we decided to create two domain-specific scales, one scale for TV and the other for mobile devices, for each of the following domains: restriction of time, coviewing, and behavioral regulation. Based on interview findings and to minimize participant burden, we kept the general term screen device for the domains of content restriction, screen use around bedtime, and screen use while eating. Item response options were never (0), sometimes (1), often (2), very often (3), and always (4). The five-point scale was chosen based on evidence suggesting that a greater number of response options is challenging for individuals with low literacy [41].

Translation: We used a team approach to translation. A bilingual/bicultural team member translated all the items. The bilingual research team then reviewed the English and Spanish language versions side by side. Applying a decentering approach [42,43], we used group discussion to determine whether any changes were needed to either language version to ensure conceptual equivalence across both languages and relevance for the Mexican American population.

Pretesting: Cognitive interviews were used to pretest items with Spanish- and English-speaking Mexican American mothers of toddlers. Trained bilingual, bicultural research assistants read each item to individual participants, asking them to express the item in their own words. Probes were utilized to assess understanding and ease of response to each item [44]. The team of investigators and research staff then discussed responses indicating problematic wording or concepts, following which some wording was altered, and some items were dropped. Cognitive interviewing continued until all items and wording were problem-free. Pretesting aimed to ensure shared conceptual meaning and easy comprehension for participants [44].

Scale administration: Items were administered as part of the larger study enrolling Mexican American mothers with toddlers aged 15–26 months old. Parenting behaviors can vary between mothers and fathers [45]. In order to eliminate variation by gender role, we focused this work on mothers since mothers, on average, spend more time in caregiving than fathers [46]. Data were collected between November 2020 and December 2023. Mothers were recruited from a safety-net health system serving the greater Denver, Colorado metropolitan area. Survey items were administered to mothers by phone, with data collected into REDCap, an electronic data capture and management tool [47]. Oral administration of items was selected due to the prevalence of literacy challenges in Latino communities [48,49]. Due to the ongoing COVID-19 pandemic, phone administration was utilized instead of in-person administration in order to minimize any transmission risks.

Measures: The parenting practice items remaining after the pretesting process were administered to all participants, except that the TV- and mobile-specific parenting practice items were only administered to participants who said the focal child had used the device type. Demographic information was also collected. This included child age and sex, as well as maternal education level, partnership status (married/partnered or not), and age. We also administered the Bidimensional Acculturation Scale for Hispanics, which consists of two scales: Hispanic acculturation (12 items, α = 0.93) and non-Hispanic acculturation (12 items, α = 0.97) [50]. Scales measure acculturation to each cultural domain (range 1–4).

Analysis: The full sample of mothers (*n* = 384) who completed the study was randomly split into two subsamples, stratified based on partner participation, with measurement evaluation conducted using exploratory factor analysis (EFA) (*n* = 192) and tested using confirmatory factor analysis (CFA) (*n* = 192). Separate EFA models were estimated for each construct using maximum likelihood estimation and promax rotation. We calculated 9 separate EFAs, with 3 constructs specific to TV use (Restrict TV Time; Coview TV; Behavioral Regulation with TV), 3 constructs specific to mobile use (Restrict Mobile Device Time; Coview Mobile Devices; Behavioral Regulation with Mobile Devices), and 3 non-device-specific constructs (Restrict Screen Content; Allow Screen Use Around Sleep; Allow Screen Use While Eating). Items with wording in the opposite direction from others were reverse-coded prior to modeling. The exact sample size is provided in the EFA results tables. Sample sizes reflect the fact that there was a small amount of missing data at the item level, and mobile device-specific scales were limited to the *n* = 161 in the EFA sample who reported allowing their child access to mobile devices (*n* = 305 of the total sample allowed access to mobile devices). Models with 1–4 factors were evaluated. The final model was selected based on the observed scree plot of eigenvalues, model interpretability, and model convergence. Single-factor models were retained for each of the 9 constructs, and items with factor loadings < 0.35 were removed.

Following this, CFA models were estimated as 9 separate one-factor models using items retained from EFA. CFA model fit was assessed using the comparative fit index (CFI ≥ 0.90) and root mean square error of approximation (RMSEA ≤ 0.08). In the event of CFA misfit, modification indices were considered, resulting in correlated residual variance values among specific items with shared variance beyond the latent factor.

Following CFA modeling, Cronbach’s alpha was calculated for each factor using the full sample of *n* = 384 (or *n* = 305 for mobile-specific scales). Scale scores were created using the items of each respective construct and were examined in relation to one another using Spearman’s correlations due to the score distributions. Scale scores were also examined in relation to demographic variables using Spearman’s correlations. Analyses were completed using SAS Version 9.4 (EFA and correlations) and MPlus Version 8.6 (CFA).

## 3. Results

The sample characteristics are presented in Table 1 for the total sample and EFA and CFA subsamples. Overall, participants were, on average, 31.4 years old (SD = 6.0), most were partnered (85%), and the mean education level was 11.6 years (SD = 2.5). Over three-quarters (77%) were interviewed in Spanish. The mean focal child age was 21.2 months (SD = 3.1).

The findings from the EFA analysis are included in Table 2, Table 3 and Table 4 for the TV-specific scales, mobile device-specific scales, and non-device-specific scales, respectively. The CFA findings are presented in Table 5.

### 3.1. TV-Specific Scales

Restrict TV Time: Following EFA, two items with loadings < 0.35 were dropped, resulting in an 8-item scale (Table 2). Fit statistics from the original CFA model indicated inadequate fit, which improved substantially after allowing two residual correlations (Table 5). Cronbach’s alpha was good (α = 0.82; Table 6) for the resulting 8-item scale. *Coview TV*: All 10 items were retained based on EFA findings (Table 2). CFA model fit statistics supported this 10-item scale (Table 5), and alpha was good (α = 0.86; Table 6). Behavioral Regulation with TV: One item was dropped following EFA, resulting in a 12-item scale (Table 2). CFA analysis showed a good fit after allowing four residual correlations (Table 5). The final scale had good internal consistency (α = 0.90; Table 6).

### 3.2. Mobile-Specific Scales

Restrict Mobile Device Time: EFA loadings indicated two items should be dropped, leaving an 8-item scale (Table 3). CFA supported the scale with two correlated residuals (α = 0.84, Table 5). Cronbach’s alpha was good (α = 0.84, Table 6) for the resulting 8-item scale. *Coview Mobile Device:* Based on EFA loadings, all 10 items were retained (Table 3), and the CFA model fit statistics supported this 10-item scale with two correlated residuals (Table 5). The alpha was good (α = 0.87, Table 6). Behavioral Regulation with Mobile Devices: One item was dropped following EFA, resulting in a 16-item scale (Table 3). CFA analysis of the resulting scale showed good fit, though only after allowing eight correlated residuals (Table 5). Cronbach’s alpha for the final scale was 0.91 (Table 6).

### 3.3. Non-Device Specific Scales

Restrict screen content: EFA loadings indicated two items should be dropped, resulting in an 8-item scale (Table 4). Model fit statistics from the CFA supported the 8-item scale with one correlated residual (Table 5). The alpha was good (α = 0.83, Table 6). Allow Screen Use Around Sleep: Two items were dropped based on the EFA, leaving six remaining items (Table 4). CFA findings supported the six-item scale (Table 5), and internal consistency reliability was good (α = 0.83, Table 6). Allow Screen Use While Eating: EFA indicated all six items should be retained (Table 4). CFA findings supported this scale with one correlated residual (Table 5), and the alpha was good (α= 0.81, Table 6).

### 3.4. Correlations Between Parenting Practice Scales

Some correlations between scales were significant (Table 6). The highest correlations were between TV and mobile device-specific measures. Restrict TV Time was highly correlated with Restrict Mobile Device Time (ρ = 0.70, *p* < 0.001). Similarly, there were high correlations between the TV-specific and mobile device-specific measures for Coviewing (ρ = 0.46, *p* < 0.001) and Behavioral Regulation (ρ = 0.65, *p* < 0.001). Behavioral Regulation with TV and with Mobile Devices were also both correlated with Allowing Screen Use Around Sleep (ρ = 0.35, *p*< 0.001; ρ= 0.35, *p*< 0.001) and Allowing Screen Use While Eating (ρ = 0.36, *p*< 0.001; ρ = 0.40, *p*< 0.001), respectively.

### 3.5. Correlations with Demographic Factors

Multiple demographic factors were correlated with the parenting practice scales (Table 7). Across scales, Hispanic acculturation, maternal age, and child age correlated with the highest number of scales. Higher Hispanic acculturation was related to a greater restriction of TV and mobile device time, coviewing of TV and mobile devices, and restriction of content, whereas higher non-Hispanic acculturation was associated with less coviewing of TV and mobile devices. Older mothers reported more restriction of TV and mobile device time. Older mothers also reported using less behavioral regulation with TV and mobile devices and allowing less screen use around eating. Mothers with older children used less restriction of TV and mobile device time and less restriction of content, and engaged in greater behavioral regulation with mobile devices.

## 4. Discussion

This study employed a mixed methods approach to develop age-specific and culturally-relevant scales of screen-related parenting practices, addressing the need for rigorously developed and evaluated measures of parenting practices in the toddler age group. Together, these measures constitute the most comprehensive measurement, to date, of screen-related parenting practices in toddlers. Moreover, by offering device-specific scales, these measures offer the opportunity for a more nuanced understanding of parenting across screen devices. All scales showed good internal consistency reliability. The development of these measures in a Mexican American sample is critical to enhance the understanding of toddler screen use in this population. The scales have strong potential to improve our understanding of parental socialization of toddlers related to screen use, and the impact on both toddler screen use and toddler health in Mexican American communities. Given that approximately 15% of children in the US are of Mexican heritage [37,38], this work applies to a large group of children.

The scales developed in this study reflect the domains of numerous parenting practices discussed in screen use guidelines published by professional organizations, including the American Academy of Pediatrics [52,53,54]. Hence, these scales offer the opportunity to evaluate the role of parenting in shaping screen use in this age group, as well as the relationship of screen-related parenting with toddler developmental and health outcomes. Over the last decade, there have been increased concerns regarding the use of screen devices for behavioral regulation in early childhood, suggesting that such practices may impact toddler development in terms of critical skills, such as self-regulation [10]. The two scales we developed, reflecting behavioral regulation with screen devices, offer the opportunity to examine such parenting practices and their relationship with child development. In addition, as experts have called for more focus on how content viewed on screens might affect children [55], the Restrict Screen Content scale will help investigators to better understand parenting in this domain. Moreover, the context-specific parenting practices related to sleep and eating may allow investigators to increase our understanding of how mothers shape toddler eating and sleeping habits, and their contribution to toddler dietary intake and sleep quality.

The device-specific measures for three domains of parenting—restriction, coviewing, and behavioral management—reflect the possibility that some mothers may vary their parenting by screen device type. These scales are an advancement of measurement in this field, meeting the call from experts for more nuanced measurement [9,56,57,58]. While items across domains for the two device types are similar, the mobile device scales capture practices relevant to the use of mobile devices across multiple settings, including away from home. The similarity in items between the TV and mobile device-specific scales may have contributed to the high correlations for each of the domains. The high correlations also suggest that overall parenting is fairly consistent across device types. Nevertheless, the separate scales offer the opportunity to evaluate device-specific parenting and its relationship with toddlers’ device-specific screen use behaviors, as well as health and developmental outcomes in toddlers. Moreover, investigators focusing on a single type of device may find these scales useful. Altogether, the device-specific scales offer an important advance in the measurement of screen-related parenting practices.

A true strength of this study is the use of the culturally-based approach to develop these measures. Latino children face disparities in childhood obesity and sleep quality [59,60,61,62,63], both of which are related to screen use [64,65], and yet there are gaps in our understanding of parenting related to screen use in Latino families [40]. These measures are available in both English and Spanish, based on rigorous pretesting to ensure consistent meaning between language versions. These scales can be used to help investigators understand screen use in this population and, importantly, in turn, to design effective interventions for this population. Of note, higher Hispanic acculturation was related to higher restriction of TV and mobile device time, more coviewing of TV and mobile devices, and more restriction of screen content, whereas higher non-Hispanic acculturation was related to less coviewing of TV and mobile devices. In our previous study with Mexican American mothers of preschoolers, we found that non-Hispanic acculturation was associated with more coviewing of TV but did not find an association with restriction of TV time or behavioral regulation with TV [28]. While these are simple correlations, these findings suggest the need for further evaluation of contextual and cultural factors within this population that may be associated with screen-related parenting practices.

Demographic factors are commonly associated with parenting practices [66]. In this study, older maternal age was associated with increased endorsement of restriction of TV and mobile device time and less use of behavioral regulation with TV and mobile devices. Evidence in other fields suggests that older maternal age is associated with parenting behaviors that promote child well-being [67]. While toddler sex was not related to any of the parenting practices, toddler age was associated with several parenting practices, even within the narrow 12-month age window of the sample. Older toddler age was associated with less maternal restriction of TV and mobile device time, more maternal behavioral regulation with mobile devices, and less maternal restriction of screen content. Although the toddler age range is narrow, parenting practices are often in the process of being established as children develop and seek more independence. The decreased limits on time and content with increasing age and increased use of mobile devices for behavioral management may reflect parental response to these developmental changes.

This study has both strengths and limitations. As previously mentioned, a strength of this work is the mixed methods approach. Moreover, the parenting practice scales address the need for screen-related measures specific to the toddler period, which is a time of increasing child desire for independence, with rapid development in language and mobility, such that parenting has unique challenges. A few limitations warrant mention. While it is a strength that the measures are available in both English and Spanish, we were unable to evaluate whether measurement invariance exists across the two languages, because the majority of the sample was interviewed in Spanish. Additionally, this work was conducted in one geographic area with a sample recruited from a safety-net clinic system, and thus, further work is needed to understand how these scales perform in other populations. Whether these scales perform similarly in fathers must also be considered. However, we did not identify any differences in screen-related parenting practices by parental role (mother, father) in our recent qualitative study enrolling Mexican American mothers and fathers with toddlers [30]. Finally, additional work to evaluate the validity of these measures would be beneficial.

## 5. Conclusions

The ever-growing reliance on screens in everyday life emphasizes the need to promote healthy screen use in daily life, balancing both the benefits and the risks [52]. Parents are the main socializers of young children, and thus, we must understand how they shape young children’s screen use. The measures developed in this study offer a way to evaluate the use and impact of screen-related parenting practices in Mexican American families with toddlers. The use of these measures will enable investigators to identify relationships among parenting practices, screen use, and child well-being. The findings of such work will support the design of effective early childhood interventions, promoting healthy screen use.

## Figures and Tables

**Table 1:** Characteristics of a sample of Mexican American mothers with toddlers aged 15–26 months (*n* = 384) *.

	Total Sample (*n* = 384)	EFA (*n* = 192)	CFA (*n* = 192)
Characteristic	Mean (SD), Median (IQR) ^†^, % (*n*)	Mean (SD), Median (IQR) ^†^, % (*n*)	Mean (SD), Median (IQR)^†^, % (*n*)
Mother			
Education (years)	11.6 (2.5)	11.4 (2.6)	11.8 (2.4)
Partnered (%)	85% (*n* = 328)	88% (*n* = 168)	83% (*n* = 160)
Age (years)	31.4 (6.0)	31.1 (6.1)	31.8 (5.8)
Acculturation: non-Hispanic	2.3 (1.7–3.2)	2.2 (1.6–3.3)	2.3 (1.7–3.1)
Acculturation: Hispanic	3.6 (3.3–3.9)	3.5 (3.3–3.9)	3.7 (3.3–3.9)
Language of interview: Spanish	77% (*n* = 268)	77% (*n* = 148)	77% (*n* = 148)
Child			
Age (months)	21.2 (3.1)	21.2 (3.1)	21.1 (3.2)
Sex (% male)	49% (*n* = 189)	52% (*n* = 100)	46% (*n* = 89)
Ever viewed TV	98% (*n* = 377)	98% (*n* = 189)	98% (*n* = 188)
Ever used mobile device	79% (*n* = 305)	84% (*n* = 161)	75% (*n* = 144)

EFA = exploratory factor analysis; CFA = confirmatory factor analysis. * Some of the above descriptive findings have already been published [51]. ^†^ Mean and standard deviation (SD) presented for approximately normally distributed variables; median and interquartile range (IQR) presented for skewed variables.

**Table 2:** Exploratory factor analysis (EFA) loadings for TV-specific measures: Restrict TV Time, Coview TV, and Behavioral Regulation with TV *.

Order **	Items	EFA Factor Loadings
	How often do you…	
Restrict TV Time (*n* = 188)		
10	control how much time (CHILD) watches TV each time he/she is using it?	0.74
7	set a time limit on TV time before (CHILD) starts to use it?	0.72
9	set a schedule of what times (CHILD) can watch TV?	0.64
4	control the amount of time (CHILD) can watch TV overall?	0.63
6	keep track of the amount of time (CHILD) watches TV?	0.61
8	set a timer to limit the amount of time that (CHILD) can watch TV?	0.52
1	control when (CHILD) can watch TV?	0.48
3	encourage (CHILD) to play with other things instead of watching TV?	0.40
Dropped		
2	^†^ let (CHILD) decide how long s/he watches TV?	0.02
5	^†^ let (CHILD) watch TV until s/he gets bored?	−0.04
Coview TV (*n* =189)		
6	watch TV with (CHILD) to learn things together?	0.75
5	watch a favorite program TV with (CHILD)?	0.74
8	view a show on TV with (CHILD) that you both like?	0.71
3	watch TV with (CHILD) just to be together?	0.69
9	watch TV with (CHILD) just for fun?	0.66
1	watch TV with (CHILD)?	0.63
7	laugh with (CHILD) about things you see on TV together?	0.63
10	watch TV together as a family?	0.55
2	sing the songs from the TV programs you are watching with (CHILD)?	0.53
4	watch TV WITH (CHILD) because s/he asked you to?	0.52
Behavioral Regulation with TV (*n* =189)		
1	have (CHILD) watch TV to calm him/her down?	0.81
2	have (CHILD) watch TV when s/he cannot sit still?	0.76
5	allow (CHILD) to watch TV to keep him/her calm?	0.74
3	have (CHILD) watch TV when you need to get things done?	0.73
11	have (CHILD) watch TV when s/he is crying?	0.69
8	allow (CHILD) to watch TV to keep him/her quiet?	0.68
4	have (CHILD) watch TV when s/he is misbehaving?	0.66
6	have (CHILD) watch TV when you are cleaning?	0.65
10	allow (CHILD) to watch TV to keep him/her safe when inside the house (like safe from cleaning products and knives)?	0.63
9	have (CHILD) watch TV when you are cooking?	0.61
13	have (CHILD) watch TV when you are in the bathroom?	0.52
12	reward (CHILD)’s good behavior with time watching TV?	0.36
Dropped		
7	turn off the TV when (CHILD) misbehaves?	−0.02

Note: Scales can be used for research purposes if this paper is cited and corresponding author is notified prior to use. A Spanish-language version is available from the author. (CHILD) should be replaced with focal child’s name. * Some items similar to items in other measures [17,28]. ** The numbering here reflects the order of administration during data collection. ^†^ Reverse coded.

**Table 3:** Exploratory factor analysis (EFA) loadings for mobile device-specific measures: Restrict Mobile Device Time, Coview Mobile Devices, and Behavioral Regulation with Mobile Devices *.

Order **	Items	EFA Factor Loadings
	How often do you…	
Restrict Mobile Device Time (*n* =160)		
6	keep track of the amount of time (CHILD) uses mobile devices?	0.75
10	control how much time (CHILD) uses a mobile device each time he/she is using it?	0.72
4	control the amount of time (CHILD) can use mobile devices overall?	0.71
7	set a time limit on mobile device use before (CHILD) starts to use it?	0.68
1	control when (CHILD) uses mobile devices?	0.64
9	set a schedule of what times (CHILD) can use mobile devices?	0.56
8	set a timer to limit the amount of time that (CHILD) can use a mobile device?	0.49
3	encourage (CHILD) to play with other things instead of using mobile devices?	0.47
Dropped		
5	^†^ let (CHILD) use mobile devices until s/he gets bored?	0.09
2	^†^ let (CHILD) decide how long s/he uses a mobile device?	0.01
Coview Mobile Devices (*n* =161)		
8	view a show or a game on a mobile device with (CHILD) that you both like?	0.76
5	watch a favorite program on a mobile device with (CHILD)?	0.73
6	use a mobile device with (CHILD) to learn things together?	0.73
3	use a mobile device with (CHILD) just to be together?	0.68
7	laugh with (CHILD) about things you see on a mobile device together?	0.67
9	use a mobile device with (CHILD) just for fun?	0.59
2	sing the songs from the programs you are watching on a mobile device with (CHILD)?	0.54
10	use a mobile device together as a family?	0.46
1	use a mobile device with (CHILD)?	0.41
4	use a mobile device WITH (CHILD) because s/he asked you to?	0.35
Behavioral Regulation with Mobile Devices (*n* = 160)		
2	have (CHILD) use a mobile device when s/he cannot sit still?	0.78
6	allow (CHILD) to use a mobile device to keep him/her calm?	0.76
1	have (CHILD) use a mobile device to calm him/her down?	0.76
3	have (CHILD) use a mobile device when you need to get things done?	0.76
7	have (CHILD) use a mobile device when you are cleaning?	0.72
14	have (CHILD) use a mobile device when s/he is crying?	0.66
10	allow (CHILD) to use a mobile device to keep him/her quiet?	0.65
11	have (CHILD) use a mobile device when you are cooking?	0.64
16	have (CHILD) use a mobile device when you are in the bathroom?	0.64
12	have (CHILD) use a mobile device when you are in a store together?	0.62
4	have (CHILD) use a mobile device when you are in the car together?	0.60
8	have (CHILD) use a mobile device when you are doing errands with him/her?	0.60
13	allow (CHILD) to use a mobile device to keep him/her safe when inside the house? (like safe from cleaning products and knives)	0.55
17	have (CHILD) use a mobile device when you are in a restaurant together?	0.54
15	reward (CHILD)’s good behavior with time using a mobile device?	0.46
5	have (CHILD) use a mobile device when s/he is misbehaving?	0.43
Dropped		
9	take away or turn off a mobile device when (CHILD) misbehaves?	0.05

Note: Scales can be used for research purposes if this paper is cited and corresponding author is notified prior to use. A Spanish-language version is available from the author. (CHILD) should be replaced with focal child’s name. * Some items similar to items in other measures [17,28]. ** The numbering here reflects the order of administration during data collection. ^†^ Reverse coded.

**Table 4:** Exploratory factor analysis (EFA) loadings for non-device-specific parenting practice measures: Restrict Screen Content, Allow Screen Use Around Sleep, Allow Screen Use While Eating *.

Order **	Items	EFA Factor Loadings
	**How often do you…**	
Restrict Screen Content (*n* = 192)		
5	pay attention to what (CHILD) is looking at on a screen device?	0.79
8	make sure you know the exact program (CHILD) is viewing?	0.70
2	make sure (CHILD) uses screen devices near you so you can monitor what s/he is watching?	0.65
6	control what (CHILD) watches on screen devices?	0.60
4	turn off the screen device if (CHILD) is watching something inappropriate?	0.54
7	watch everything (CHILD) sees on screen devices before she/he watches it?	0.48
3	limit (CHILD) to watching specific program?	0.44
1	change the program when (CHILD) is watching something inappropriate?	0.43
Dropped		
9	^†^ let (CHILD) decide what to watch on screen devices?	0.15
10	use controls on screen devices to limit what (CHILD) can see?	0.15
Allow Screen Use Around Sleep **(***n* = 192)		
3	let (CHILD) use/watch a screen device in bed at bedtime?	0.81
2	use a screen device to help (CHILD) fall asleep?	0.80
6	let (CHILD) fall asleep while he/she uses a screen device?	0.79
1	allow (CHILD) to use screen devices before bed?	0.72
5	have (CHILD) use a screen device if s/he can’t sleep at night?	0.56
4	have (CHILD) sleep all night with a screen device on?	0.35
Dropped		
8	^†^ limit what kinds of programs (CHILD) can watch before bed?	0.14
7	^†^ limit how much (CHILD) uses screen devices before bed?	0.08
Allow Screen Use While Eating (*n* = 192)		
5	is there a screen device on when (CHILD) eats?	0.83
4	have a TV on in the background when (CHILD) is eating?	0.82
3	use a screen device to get (CHILD) to eat?	0.67
1	allow (CHILD) to use a screen device during meals?	0.63
2	^†^ make sure all screen devices are off at mealtime?	0.55
6	allow (CHILD) to use a screen device while he/she eats something in between meals?	0.42

Note: Scales can be used for research purposes if this paper is cited and corresponding author is notified prior to use. A Spanish-language version is available from the author. (CHILD) should be replaced with focal child’s name. * Some items similar to items in other measures [17,28]. ** The numbering here reflects the order of administration during data collection. ^†^ Reverse coded.

**Table 5:** Model fit statistics for confirmatory factor analysis (CFA) for 9 screen-related parenting practice scales.

	**Model Fit Statistics**		
**Scale**	**CFI**	**RMSEA** **(90% CI)**	**SRMR**
Restrict TV Time			
Original Model	0.84	0.14 (0.12, 0.17)	0.07
With correlated residuals Items *: 1,3; 6,7	0.92	0.11 (0.08, 0.14)	0.06
Coview TV			
Original Model	0.98	0.04 (0.00, 0.07)	0.04
Behavioral Regulation with TV			
Original Model	0.80	0.15 (0.13,0.16)	0.08
With correlated residuals Items *: 1,9; 1,10; 6,8; 8,9	0.91	0.10 (0.09,0.12)	0.07
Restrict Mobile Device Time			
Original model	0.79	0.18 (0.15, 0.22)	0.09
With correlated residuals Items *: 1,3; 6,7	0.91	0.12 (0.09, 0.16)	0.07
Coview Mobile Devices			
Original model	0.88	0.13 (0.10, 0.15)	0.06
With correlated residuals Items *: 1,7; 3,10	0.91	0.11 (0.08, 0.14)	0.06
Behavioral Regulation with Mobile Devices			
Original model	0.73	0.15 (0.13, 0.16)	0.09
With correlated residuals Items *: 1,2; 7,10; 8,11; 9,13; 10,12; 10,16; 11,16; 13,16	0.90	0.09 (0.08, 0.11)	0.06
Restrict Screen Content			
Original model	0.89	0.13 (0.11, 0.16)	0.07
With correlated residuals Items *: 5,6	0.95	0.09 (0.06, 0.13)	0.05
Allow Screen Use Around Sleep			
Original Model	0.95	0.13 (0.09, 0.17)	0.05
Allow Screen Use While Eating			
Original model	0.78	0.23 (0.20, 0.28)	0.09
With correlated residuals Items *: 4,5	0.94	0.13 (0.09, 0.18)	0.06

CFI: comparative fit index; RMSEA: root mean square error of approximation; SMSR: standardized root mean square residual. * Item numbers reflect the item numbering for each scale in Table 2, Table 3 and Table 4.

**Table 6:** Means, alphas, and Spearman correlations for screen-related parenting practice scales (*n* = 305–384).

	Parenting Practice Scales								
	Restrict TV Time	Coview TV	Behavioral Regulation With TV	Restrict Mobile Device Time	Coview Mobile Devices	Behavioral Regulation with Mobile Devices	Restrict Screen Content	Allow Screen Use Around Sleep	Allow Screen Use While Eating
Scale Alpha	0.82	0.86	0.90	0.84	0.87	0.91	0.83	0.83	0.81
Scale mean (SD)	2.31 (0.87)	2.00 (0.79)	0.98 (0.61)	2.48 (0.95)	1.29 (0.68)	0.77 (0.51)	3.46 (0.67)	1.01 (0.77)	0.44 (0.58)
Restrict TV Time	1.00 **	0.16 **	−0.31 ***	0.70 ***	0.11	−0.28 ***	0.36 ***	−0.31 ***	−0.31 ***
Coview TV		1.00 **	0.25 ***	0.14 *	0.46 ***	0.14 *	0.15 **	0.04	0.11 *
Behavioral Regulation With TV			1.00 **	−0.25 ***	0.25 ***	0.65 ***	−0.21 ***	0.35 ***	0.36 ***
Restrict Mobile Device Time				1.00 **	0.06	−0.36 ***	0.37 ***	−0.27 ***	−0.28 ***
Coview Mobile Devices					1.00 **	0.36 ***	−0.01	0.08	0.19 ***
Behavioral Regulation With Mobile Device						1.00 **	−0.24 ***	0.35 ***	0.40 ***
Restrict Screen Content							1.00 **	−0.18 ***	−0.19 ***
Allow Screen Use Around Sleep								1.00 **	0.31 ***
Allow Screen Use While Eating									1.00 **

* *p* < 0.05, ** *p* < 0.01, *** *p* < 0.001.

**Table 7:** Screen-related parenting practice scales: Spearman correlations with demographic factors (*n* = 305–384).

Demographic Variables	Restrict TV Time	Coview TV	Behavioral Regulation With TV	Restrict Mobile Device Time	Coview Mobile Devices	Behavioral Regulation with Mobile Devices	Restrict Screen Content	Allow Screen Use Around Sleep	Allow Screen Use While Eating
**Mother**									
Education (years)	−0.10	−0.09	−0.06	−0.12 *	−0.05	−0.01	−0.05	0.02	0.20 ***
Partnered	0.05	0.08	−0.02	0.07	−0.09	−0.08	0.03	−0.01	0.02
Age (years)	0.12 *	−0.03	−0.19 ***	0.13 *	0.02	−0.16 **	0.09	−0.07	−0.17 ***
Acculturation: non-Hispanic	−0.08	−0.11 *	−0.01	−0.10	−0.14 *	−0.04	−0.06	0.06	0.07
Acculturation: Hispanic	0.15 **	0.17 **	0.01	0.12*	0.16 **	0.03	0.12*	−0.04	−0.07
Language of interview: Spanish	−0.11 *	−0.08	0.03	−0.10	−0.07	−0.01	−0.09	0.14 **	0.10
**Child**									
Age (months)	−0.15 **	0.00	0.07	−0.1 6**	0.06	0.19 **	−0.13 **	0.08	0.06
Sex (male)	−0.06	−0.01	0.01	−0.02	0.00	0.08	0.07	0.00	−0.05

* *p* < 0.05, ** *p* < 0.01, *** *p* < 0.001.

## Data Availability

The data analyzed for this article are not promptly available because a data sharing agreement is required. Requests to access the dataset should be directed to Darcy Thompson, darcy.thompson@cuanschutz.edu.
